# Extended resection including adjacent organs and Ki-67 labeling index are prognostic factors in patients with retroperitoneal soft tissue sarcomas

**DOI:** 10.1186/s12957-016-0810-z

**Published:** 2016-02-24

**Authors:** Yosuke Morizawa, Makito Miyake, Keiji Shimada, Shunta Hori, Yoshihiro Tatsumi, Yasushi Nakai, Satoshi Anai, Nobumichi Tanaka, Noboru Konishi, Kiyohide Fujimoto

**Affiliations:** Department of Urology, Nara Medical University, 840 Shijo-cho, Nara, 634-8522 Japan; Department of Pathology, Nara Medical University, Nara, Japan

**Keywords:** Extended resection, Ki-67, Prognostic factor, Retroperitoneal, Soft tissue sarcoma

## Abstract

**Background:**

Because retroperitoneal soft tissue sarcomas (RPS) are extremely rare, there is a significant lack of clinicopathologic information to optimize the treatment strategy. The aim of this study was to evaluate the prognostic factors in RPS, with particular focus on the Ki-67 labeling index (LI).

**Methods:**

We included the data from a total of 23 patients who received treatment for primary RPS at a single center. The variables analyzed in this study included tumor size, histological type, malignancy grade, necrosis, mitosis, and Ki-67 LI. Kaplan-Meier and Cox proportional regression analyses of overall survival (OS) were performed to identify significant prognostic variables.

**Results:**

Of the 23 patients who underwent surgical resection, 9 (39 %) underwent simple resection of the tumor and 14 (61 %) extended resection including the adjacent organs. In the univariate analysis, a simple tumor resection and a high Ki-67 LI were associated with shorter OS. The multivariate analysis revealed that simple tumor resection and a high Ki-67 LI were independent negative prognostic factors for OS.

**Conclusions:**

Our results suggested that combined resection of RPS and its adjacent organs improved OS. Pathologically, a high Ki-67 LI was significantly associated with negative prognosis.

## Background

Retroperitoneal soft tissue sarcomas (RPS) are uncommon tumors, accounting for approximately 12 % of soft tissue sarcomas [1]. The mean annual incidence is approximately 2.7 per 100,000 [2], and the 5-year survival is approximately 50 % [3]. Local recurrence is common (41–68 %), and metastases occur in one third of the patients after surgical resection [3]. Local recurrence is the leading cause of death in patients with RPS. Surgery is currently the only modality that offers a chance of cure.

Extended resection including the adjacent organs is suggested regardless of the presence of neoplastic infiltration [4–7]. On the other hand, the surgery for RPS presents specific challenges due to the location in a complex space surrounded by vital structures and visceral organs, and an extended surgery is not practical in all cases [8–10]. Adjuvant therapy such as radiotherapy and/or systemic chemotherapy is usually considered because the risk of local recurrence after surgery alone is relatively high.

Prognostic factors in RPS are important for selecting appropriate therapies and follow-up after surgical resection. The currently used prognostic factors include age, tumor size, histopathological type, malignancy grade, and positive resection margins; these are combined into different prognostic systems [3, 5, 11–15]. To improve the prognostic accuracy, researchers have been trying to incorporate an assessment of the biological features, such as proliferation activity, into the prediction tools for disease prognosis. The Ki-67 labeling index (LI), assessed with immunohistochemical staining using a mouse monoclonal antibody named MIB-1 clone, and based on the proportion of Ki-67-positive tumor cells, is known to be useful for objective histopathological evaluation of the tumor proliferation activity. A high Ki-67 LI is associated with poor prognosis in soft tissue sarcomas [16, 17]. Ki-67 has also been recognized as essential for diagnosing tumor grade. The purpose of this study was to evaluate the currently used prognostic factor and to clarify the prognostic significance of Ki-67 in RPS in a Japanese single-center cohort.

## Methods

### Patients and data collection

The Nara Medical University institutional review board approved this study, and all participants provided informed consent. This is a retrospective study of 26 cases with RPS, treated between January 2002 and January 2014 at Nara Medical University. In three patients, only needle biopsy was carried out for the verification of pathology, because significant comorbidities and/or advanced stage made them unsuitable for a radical resection. One of these patients was treated with palliative care alone, one with radiation therapy, and one with chemotherapy. These three patients were excluded, leaving 23 cases. The type of surgery was categorized into two groups: simple resection (surgical resection of the tumor mass only) and extended resection (surgical resection of the tumor mass and the adjacent organs). All the hematoxylin and eosin (H&E)-stained specimens were re-evaluated for diagnosis by two experienced pathologists (KS and KN), following the World Health Organization (WHO) criteria for classification of soft tissue tumors [18]. The tumor variables analyzed included tumor size, histological type, malignancy grade [19], presence of necrosis, and cell mitosis.

### Calculation of the Ki-67 labeling index

Immunohistochemical (IHC) staining for Ki-67 was performed with a streptavidin-biotin (SAB) complex method using the Histofine SAB-PO kit (Nichirei Co., Tokyo, Japan) according to the manufacturer’s directions. For antigen retrieval, the sections were routinely autoclaved for 10 min in 0.01 M citrate buffer (pH 6.0). The primary antibody was monoclonal mouse anti-Ki-67 antigen (clone MIB-1, Dako, Japan), ready to use at room temperature for 30 min. The sections were counterstained with Meyer’s hematoxylin and mounted with Malinol and were examined alongside H&E-stained specimens, to identify the precise locations of the lesions. Five independent areas in the sections were selected with a high-power field (×400) and saved as pictures. IHC evaluation was carried out by two investigators (YM and YT). Evaluation was performed blindly without knowledge of the patients’ outcome and other clinicopathologic characteristics. The percentages of positive cells (Ki-67 LI) were calculated. The cutoff point was determined by a receiver operating characteristics (ROC) curve for tumor recurrence.

### Statistical analysis

The primary endpoint was overall survival (OS). The cutoff date of the last follow-up was December 31, 2015. The OS was computed from the date of primary tumor resection to the date of death or the last follow-up and examined using the Kaplan-Meier method. Univariate and multivariate analyses were performed for OS using the Cox proportional hazards models. All tests were two-sided. The results of Cox model analysis are reported with relative risks and 95 % confidence intervals (CIs). IBM SPSS version 21 (SPSS Inc., Chicago, IL) and PRISM software version 5.00 (GraphPad Software, Inc., San Diego, CA) were used for statistical analyses and data plotting, respectively. A *P* value of <0.05 was considered statistically significant.

## Results

### Patient characteristics and clinical and operative findings

The median patient age was 62.5 years (range 31–79 years). There were 15 male (65 %) and 8 female participants (35 %). The clinicopathologic variables of the patients and primary tumors are presented in Table 1. Nine received a simple resection of the tumor and 14 an extended resection including the adjacent organs. Type of surgery was decided by preoperative imaging or intraoperative examination. Ten patients underwent the extended resection because they were diagnosed as infiltration into the adjacent organs by preoperative imaging. In one of these ten patients, whose tumor infiltrated to the kidney and diaphragm, only the tumor and kidney were resected. Four patients underwent the extended resection because the surgeons suspected the infiltration by intraoperative examination. One patient who was diagnosed as infiltration into the adjacent organs performed the simple resection because resection of the adjacent organs was impossible for tumor adhesion. The main resected organs were the kidney (6/10, 60 %), colon (4/10, 40 %), and bladder (3/10, 30 %). In four patients, two adjacent organs were resected, and in two patients, three organs were resected en bloc. In all patients, there were no major perioperative complications more than grade 2 in Clavien classification.

**Table 1:** Clinicopathologic background according to the disease status

Variables	All (*n* = 23)	Simple resection (*n* = 9)	Extended resection (*n* = 14)	*P* value
Median age, years (IQR)	62 (51.5–71)	62 (42–70)	62 (52.5–71)	0.68^a^
Sex, *n* (%)				0.57^b^
Men	15 (65 %)	7 (78 %)	8 (57 %)	
Women	8 (35 %)	2 (22 %)	6 (43 %)	
Follow-up, months (IQR)	30 (18–51.5)	19 (18–33)	30.5 (24–87.5)	0.12^a^
Tumor size, mm (IQR)	77 (50–210)	80 (50–120)	75 (51–112)	0.99^a^
Symptoms positive, *n* (%)				0.87^b^
Yes	12 (52 %)	4 (44 %)	8 (57 %)	
No	11 (48 %)	5 (56 %)	6 (43 %)	
Infiltration into the adjacent organs, *n* (%)				0.01*^,b^
Yes	11 (48 %)	1 (11 %)	10 (71 %)	
No	12 (52 %)	8 (89 %)	4 (29 %)	
Margins, *n* (%)				0.94^b^
R0	4 (17 %)	2 (22 %)	2 (14 %)	
R1	17 (74 %)	6 (67 %)	11 (79 %)	
R2	2 (9 %)	1 (11 %)	1 (7 %)	
Histological type, *n* (%)				0.87^b^
Liposarcoma	12 (52 %)	4 (44 %)	8 (57 %)	
Leiomyosarcoma	4 (17 %)	2 (22 %)	2 (14 %)	
MFH	3 (14 %)	1 (11 %)	2 (14 %)	
Other	4 (17 %)	2 (22 %)	2 (14 %)	
Tumor grade, *n* (%)				0.82^b^
Grade 1	3 (14 %)	1 (11 %)	2 (14 %)	
Grade 2	4 (17 %)	2 (22 %)	2 (14 %)	
Grade 3	16 (69 %)	6 (67 %)	10 (71 %)	
Necrosis, *n* (%)				0.88^b^
Yes	17 (74 %)	7 (78 %)	10 (71 %)	
No	6 (26 %)	2 (22 %)	4 (29 %)	
Mitosis, *n* (%)				0.61^b^
1–9/10HPF	14 (61 %)	5 (56 %)	9 (64 %)	
10–20/HPF	9 (39 %)	4 (44 %)	5 (36 %)	
Ki-67 LI, *n* (%)				0.42^b^
<25 %	13 (56 %)	4 (44 %)	9 (64 %)	
≥25 %	10 (44 %)	5 (56 %)	5 (36 %)	

Simple resection: resection of only the tumor; extended resection: resection extended to adjacent organs; tumor grade: using the French Federation of Cancer Centers Sarcoma Group grading system

*IQR* interquartile range, *R0* microscopically negative margin, *R1* microscopically positive margin, *R2* gross residual margin, *MFH* malignant fibrous histiocytoma, *HPF* high-power field, *Ki-67 LI* Ki-67 labeling index

^a^Mann-Whitney test

^b^Fisher’s exact test or chi-square with Yates’ correction

* = *P* <0.05

### Pathological findings

The majority of the tumors were of high grade, and the most common histology was liposarcoma (52 %). Other histologies included leiomyosarcoma (17 %), malignant fibrous histiocytoma (MFH) (14 %), and others (synovial sarcoma, epithelioid sarcoma, Ewing’s sarcoma, and not otherwise-specified sarcoma). Histological type other than liposarcoma (non-liposarcoma), tumor necrosis, cell mitosis, and malignancy grade were used as prognostic factors for RPS. Cox analysis was performed to evaluate the association between these factors and OS. None of them was significant.

**Fig. 1:**
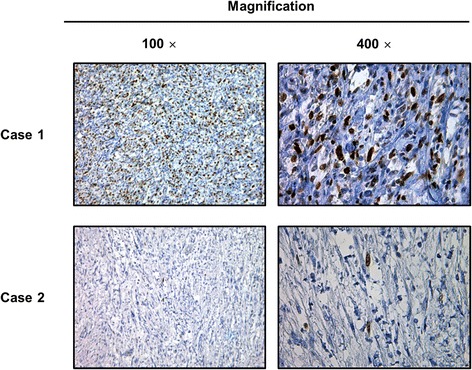
Ki-67 immunohistochemical staining of two representative cases of retroperitoneal liposarcoma. Case 1 was diagnosed with liposarcoma with a Ki-67 labeling index of 50 %. He experienced local recurrence and lung metastasis 19 months after surgical resection and died 18 months after surgical resection. Case 2 was diagnosed with liposarcoma with a Ki-67 labeling index of 2 %. He was alive without any evidence of disease recurrence at the last visit, 108 months after the surgical resection

The Ki-67 LI was evaluated in all cases (Fig. 1). The median Ki-67 LI was 25 % (range 2–53 %). The cutoff point was determined as 25 % according to the ROC curve for tumor recurrence. To evaluate the prognostic significance of the Ki-67 LI, the Kaplan-Meier and the COX analyses were performed. In univariate analysis, tumors with a Ki-67 LI of 25 % or more showed worse prognosis than those with a Ki-67 LI of less than 25 % (*P* = 0.001). Non-liposarcoma histopathology was not a negative prognostic factor compared to liposarcoma histopathology in our cohort; however, there was a significant association between a high Ki-67 LI and histological type (*P* = 0.047, median Ki-67 LI = 28.48 and 18.90 in liposarcoma and non-liposarcoma, respectively).

### Follow-up and overall survival

**Fig. 2:**
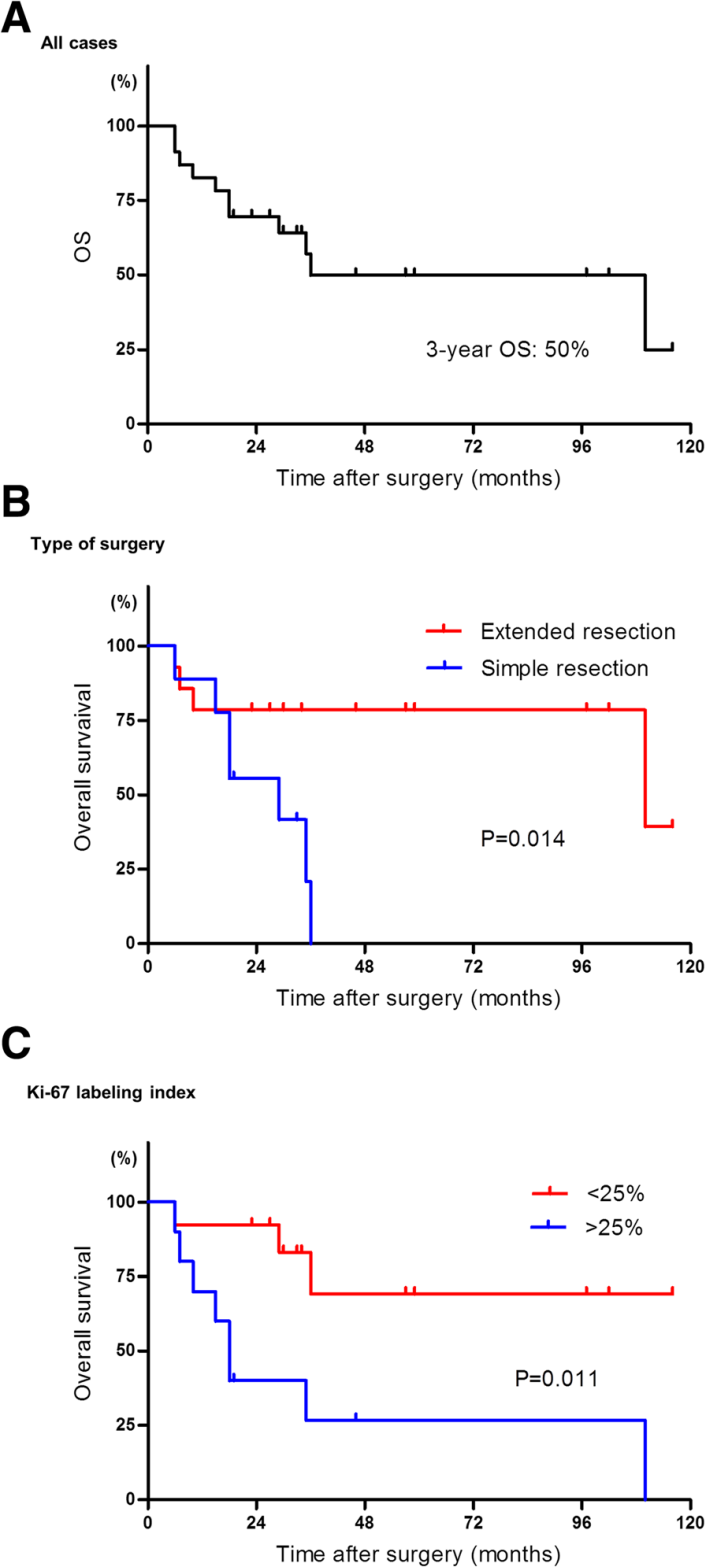
Kaplan-Meier curves of overall survival (OS) after surgical resection. **a** OS in all cases. All the events were due to tumor progression. **b** OS according to the type of surgery. The extended surgery improved OS in RPS. **c** OS according to the Ki-67 labeling index. *P* values were calculated using the log-rank test

The median follow-up period was 30 months (range 6–116 months) after surgical resection. Of the 13 patients experiencing recurrence, 6 patients experienced local recurrence in the original tumor site, whereas 7 patients developed distant metastasis. The most common site of distant metastasis was lung (in four patients). Eleven patients died of tumor progression in 6 months after recurrence. Ten patients did not present evidence of disease recurrence after surgical resection, and the median OS was 52 months. In univariate Kaplan-Meier analyses, the extended resection including the adjacent organs was associated with the probability of OS (3-year OS 78 % vs. 0 %, Fig. 2b). Ki-67 LI was associated with the probability of OS (3-year OS 69 % vs. 27 %, Fig. 2c). In multivariate Cox models, age, symptoms, tumor size, resection margins, histological type, tumor grade, and necrosis were not significant prognostic factors for OS, while the type of surgery and the Ki-67 LI were significant (Table 2). In multivariate analysis, simple resection and high Ki-67 LI were independent predictive factors for decreased OS (Table 2).

**Table 2:** Univariate and multivariate analysis of prognostic factors for overall survival

Factor	Univariate analysis			Multivariate analysis		
	HR	95 % CI	*P*	HR	95 % CI	*P*
Age (over/under 75 years old)	0.66	0.08–5.24	0.70			
Symptoms (symptomatic/asymptomatic)	2.44	0.63–9.49	0.20			
Tumor size (over 10 mm/under 10 mm)	1.49	0.45–4.95	0.52			
Type of surgery (simple/extended)	4.88	1.21–19.66	*0.026	3.80	1.25–16.59	*0.040
Surgical margins [(R1 + R2)/R0]	3.35	0.42–26.90	0.26			
Tumor grade [grade 3/(grade 1 + 2)]	1.49	0.31–7.16	0.71			
Necrosis (presence/absence)	3.05	0.38–24.46	0.29			
Histologic type (non-liposarcoma/liposarcoma)	3.82	0.98–14.89	0.054			
Ki-67 LI (≥25 %/<25 %)	4.79	1.25–18.35	*0.022	3.74	1.32–14.94	*0.049

Simple: only tumor resection; extended: resection with adjacent organs

*R0* microscopically negative margin, *R1* microscopically positive margin, *R2* gross residual margin, *HR* hazard ratio, *CI* confidence interval, *Ki-67 LI* Ki-67 labeling index, * = *P* <0.05

## Discussion

RPS is a rare neoplasm and is usually locally advanced at the time of diagnosis [3]. The mainstay of treatment is surgery. The RPS borders with the surrounding organs, including the kidney, adrenal, colon, ureter, bladder, spleen, pancreas, and psoas, are usually difficult to identify. Essentially all the macroscopic total resections consist of marginal excisions through the reactive zone surrounding the tumor; therefore, the margins are microscopically positive in the majority of the patients, and the probability of local recurrence after surgery alone is high [1, 2, 7]. In this study, the resection margin had no relevance to OS. The rate of positive resection margin (R1 or R2) was very high (83 %,19/23); nevertheless, the extended resection of adjacent organs, regardless of resection margin positivity, was a significant predictor of OS.

In contrast to the more common epithelial tumors, which develop within a single organ, RPS can infiltrate multiple surrounding organs [20]. The oncologic benefit of extended surgery is associated with a lower rate of local recurrence [6, 7]. However, the feasibility of such resection is variable, depending on the exact relationships of the tumor with other structures within the anatomical complexity of the retroperitoneum. Resection of more than three adjacent organs has been shown to be associated with a significantly higher complication rate than the resection of fewer organs [10]. In this study, resections of three adjacent organs were performed in two patients and of one or two organs in 12 patients; in all these cases, there were no major perioperative complications. In one case of tumor infiltration into multiple adjacent organs, a complete resection was not attempted. Although the extended resection of adjacent organs regardless of tumor infiltration is the gold standard [6, 7, 21], significant controversy persists regarding the benefit and associated morbidity of multi-organ resection. Some authors have proposed to perform surgery with the goal of complete macroscopic resection and to resect contiguous organs only when directly infiltrated [8–10]. Four patients who subjected to extended resection did not show tumor infiltration to the adjacent organs by diagnostic imaging, but the surgeons suspected the direct infiltration to contiguous organs by intraoperative examination. All of them did not experience recurrence.

Tumor size, malignancy grade, positive margins, and histological type are currently used as prognostic factors for RPS [3, 5, 11–15, 19]. In this study, high expression of Ki-67 was a significant predictor of OS. The Ki-67 antigen is a cell cycle-associated nuclear protein and is present in all stages of the cell cycle (G1, S, G2, and M phases) in proliferating cells but is absent in G0 and early G1 phases of cells re-entering the cell cycle [22]. The antibody against Ki-67 has been documented to be useful for the diagnosis and prognosis of some neoplasms. The Ki-67 LI is correlated with shorter disease-specific survival in soft tissue sarcomas [16, 17] and is expected to be one of the adverse prognostic factors in RPS. Because the risk of local recurrence after surgery alone is very high, adjuvant therapy should be considered [3]. Adjuvant chemotherapy or radiotherapy improved local tumor control in high-risk patients [23–25]. Therefore, the high-Ki-67 LI group represents a good candidate for adjuvant therapy.

This investigation confirmed the finding that, in patients with primary RPS, extended resection of the adjacent organs was an independent prognostic factor. However, in patients whose tumors do not infiltrate the adjacent organs according to clinical imaging, it is problematic to decide if the adjacent organs should be resected en bloc. In nine patients who subjected to simple tumor resection, eight did not show tumor infiltration to the adjacent organs by diagnostic imaging or intraoperative examination. But because seven of these eight patients experienced evidence of disease recurrence, extended resection including the adjacent organs might have been required to remove the whole tumor burden. Preoperative core needle biopsy in RPS has been reported to be useful to obtain a histological diagnosis [26]. The assessment of the Ki-67 LI by preoperative biopsy would allow selection of the appropriate operation method.

The limitations of the present study include a small sample size and the retrospective analysis. As only one patient received adjuvant chemotherapy, it was difficult to assess the importance of adjuvant therapy. Eight patients received chemotherapy or radiotherapy (chemotherapy only, 5 patients; chemotherapy + radiotherapy, 3 patients) after evidence of disease recurrence.

## Conclusions

Our results suggested that combined resection of RPS and its adjacent organs improved OS. Pathologically, a high Ki-67 LI was a significant predictor of OS.
